# Electromyography of shoulder muscles in individuals without scapular dyskinesis during closed kinetic chain exercises on stable and unstable surfaces: a systematic review and meta-analysis

**DOI:** 10.3389/fspor.2024.1385693

**Published:** 2024-05-22

**Authors:** Ramin Arghadeh, Mohammad Hossein Alizadeh, Hooman Minoonejad, Rahman Sheikhhoseini, Mojtaba Asgari, Thomas Jaitner

**Affiliations:** ^1^ Department of Sports Injury and Biomechanics, Faculty of Sport Sciences and Health, University of Tehran, Tehran, Iran; ^2^ Department of Corrective Exercises and Sports Injury, Faculty of Physical Education and Sport Sciences, Allameh Tabataba’i University, Tehran, Iran; ^3^ Institute for Sport and Sport Science, TU Dortmund University, Dortmund, Germany

**Keywords:** closed kinetic chain, dyskinesis, electromyography, push-up, shoulder

## Abstract

**Introduction:**

Unstable surfaces are commonly utilized to enhance the flexibility of the musculoskeletal system for achieving training or rehabilitation goals. However, their effects on shoulder muscle activation during various push-up (PU) exercises have not been thoroughly investigated. Therefore, the purpose of this study was to synthesize electromyography (EMG) data of shoulder muscles in individuals without scapular dyskinesis performing different PU exercises on both stable and unstable surfaces.

**Methods:**

A systematic online search was conducted in electronic databases, including Web of Science, PubMed, Scopus, and Google Scholar, up to January 16, 2024, using predefined sets of keywords. Out of the 1,971 titles and abstracts screened, 80 articles were reviewed in detail by two independent researchers to check the eligibility, of which 28 eligible studies were ultimately included. Following assessment of the quality and risk of bias, the studies were categorized based on exercises and muscle groups, and a meta-analysis using a random-effects model was performed to estimate the overall effect size.

**Results:**

The use of unstable surfaces led to a decrease in anterior deltoid activity during PU [*P* = 0.032; *I*^2^ = 91.34%; SMD = −0.630 (95% CI −1.205, −0.055)], an increase in pectoralis major activity during PU [*P* = 0.006; *I*^2^ = 63.72%; SMD = 0.282 (95% CI 0.079, 0.484)], as well as during knee PU [*P* = 0.018; *I*^2^ = 32.29%; SMD = 0.309 (95% CI 0.052, 0.565)], and an increase in triceps brachii activity during PU [*P* = 0.000; *I*^2^ = 85.05%; SMD = 0.813 (95% CI 0.457, 1.168)], knee PU [*P* = 0.000; *I*^2^ = 0.00%; SMD = 0.589 (95% CI 0.288, 0.891)], as well as during push-up plus [*P* = 0.006; *I*^2^ = 13.16%; SMD = 0.563 (95% CI 0.161, 0.965)]. However, the use of unstable surfaces did not show a significant effect on the EMG activity of the pectoralis major during push-up plus [*P* = 0.312; *I*^2^ = 22.82%; SMD = 0.207 (95% CI −0.194, 0.609)].

**Conclusions:**

Unstable surfaces can modulate muscle activity in different PU exercises, while the effects on the targeted muscles depend on the type of exercise. The findings of this review provide a framework based on the level of activity of each shoulder muscle during different PU exercises, which can help coaches, trainers, and sports therapists select the most suitable type of PU for designing training or rehabilitation programs. Particularly, the most suitable exercise for increasing anterior deltoid activity is PU on a stable surface. To concurrently increase activity of the pectoralis major and triceps brachii, adding unstable surfaces under hands during knee PU and standard PU is recommended.

**Systematic Review Registration:**

PROSPERO, identifier CRD42021268465.

## 1 Introduction

Upper limb training programs are commonly employed to increase stability, strength, and mobility while reducing the risk of injury (1). To select the most ideal exercises, Karandikar and Vargas (2) compared the concepts and clinical applications of open and closed kinetic chain exercises and concluded that a comprehensive training program should encompass a combination of these two types of exercises (2). However, closed kinetic chain exercises have garnered greater attention among sports and rehabilitation specialists due to their ability to provide increased stability and lower joint loading, which makes them more weight-bearing-tolerant (3).

Push-up and push-up plus exercises are examples of closed kinetic chain training protocols that are widely utilized in shoulder injury rehabilitation (4). The plus phase of push-up plus exercise involves full protraction of the scapular resulting from posterior thorax translation, which can be performed either independently or as a continuation of the concentric phase of traditional push-up (5). Many individuals in the initial phases of fitness or rehabilitation programs are not be able to perform push-up plus exercises with full range of motion, therefore modified push-up plus exercises, such as knee push-up plus, elbow push-up plus, and wall push-up plus, are recommended as effective and safe alternatives (6, 7). The popularity of push-up based exercises may stem from their versatility in various technical variations, ability to be performed with minimal or no equipment, and the simplicity of required skills, enabling easy adjustment of exercise intensity (8–11). Push-up exercises are used in multiple applications, including upper limb muscle recovery (especially shoulder muscles), as alternative to traditional weighted exercises for dynamic warm-up or valuable assessment tools to evaluate the upper limb performance of individuals (12–16).

While push-up are recognized as effective compound exercises (17), their proper application requires consideration of some important factors. One critical aspect of utilizing push-up exercises for assessment or strength training is the understanding of upper limb muscle activity patterns to maximize benefits, which necessitates the use of surface electromyography (EMG) to quantify muscle activity patterns (4). Several studies have investigated the EMG activity of upper limb muscles during push-up exercises and highlighted the anterior deltoid (AD), pectoralis major (PM), and triceps brachii (TB) as primary movers (8, 18–20). Additionally, the type of surface (stable or unstable) utilized to support the limbs during closed kinetic chain exercises is an important influencing factor on EMG signals (21, 22). Various unstable surface interventions have been proposed as potent means to enhance the flexibility of the musculoskeletal system without the addition of external loads due to the increased movement oscillations and enhanced neuromuscular engagement for joint stabilization (23–27).

Recently, four systematic review and meta-analysis studies attempted to compare and combine data on muscle activity during different push-up and push-up plus exercises on stable and unstable surfaces (28–31). However, in these studies, the effect of unstable surfaces was mainly assessed on the stabilizer muscles of the scapular (i.e., trapezius and serratus anterior). Considering that all shoulder muscles are relevant targets in exercise interventions and rehabilitation, it is essential to synthesize and compare data on the activity of other shoulder muscles (AD, PM, and TB) during different push-up exercises on stable and unstable surfaces in the available literature. Hence, the aim of the current study was to compare EMG activity of the primary shoulder movers during various types of push-up on stable and unstable surfaces in individuals without scapular dyskinesis.

## 2 Methods

The present meta-analysis was conducted following the Preferred Reporting Items for Systematic Reviews and Meta-Analyses (PRISMA) guidelines (32) and registered in the PROSPERO database with the identifier CRD42021268465.

### 2.1 Search strategy

A systematic online search was performed in electronic databases, including Web of Science (WOS), PubMed, Scopus, and Google Scholar, up to January 16, 2024. The search was conducted using predefined sets of keywords, including (scapul* OR shoulder OR glenohumeral OR scapulothoracic OR orientation OR protraction OR malposition OR rhythm OR dysrhythmia OR dyskines* OR dysfunction OR “sick scapul*” OR wing* OR floating OR tipp* OR tilt* OR “scapul* downward rotation syndrome” OR muscle OR muscular) AND (Electromyograph* OR “EMG” OR electromyogram OR “root mean square” OR “root-mean-square” OR “RMS” OR pattern OR recruitment OR activ* OR coactiv* OR co-activ* OR cocontract* OR co-contract* OR timing OR onset OR offset) AND (Push*-up* OR “push*up*” OR “Push* up*” OR press*-up* OR “press*up*” OR “press* up*” OR “Close* kinetic chain” OR “close* kinematic chain” OR “Close* chain”). It should be noted that our goal for the first category of keywords was to access information about the control group. We sought studies that compared the EMG activity of shoulder girdle muscles in individuals with and without scapular dyskinesis.

### 2.2 Study criteria

The review question was formulated using the PICO format (Population, Interventions, Comparators, and Outcomes) (33) as follows: Are there any differences in the EMG activity of shoulder primary movers (O) during various types of push-up exercises on stable (C) and unstable (I) surfaces in individuals without scapular dyskinesis (P)?

The studies were included if they met the following criteria: (i) included participants without shoulder abnormalities such as scapular dyskinesis (SICK scapular syndrome), scapular winging, scapular tipping, scapular downward rotation syndrome, and pain with normal shoulder joint movements, (ii) examined types of bilateral push-up and push-up plus exercises on various stable and unstable surfaces while maintaining hands and feet in contact with the base of support during the entire movement, and (iii) evaluated the EMG activity of shoulder primary mover muscles. It is worth noting that our research only included studies whose full texts were published in English-language journals with a peer-review process.

Studies that solely evaluated shoulder muscle activity on either a stable or unstable surface or focused on long-term adaptation protocols and assessed muscle activity under fatigue conditions were excluded. Moreover, all review articles, case reports, conference papers, and studies lacking sufficient information on shoulder muscle EMG activity were excluded.

### 2.3 Study selection

After removing duplicates, the remaining articles were independently screened by two researchers in two stages. In the first stage, the articles were screened based on titles and abstracts, and in the second stage, full-text articles were thoroughly evaluated based on the inclusion criteria. Any discrepancies in the selection of articles were resolved through discussion between the two researchers or, if necessary, by consulting a third researcher.

### 2.4 Data extraction

To minimize errors and bias, data were extracted from the final articles based on the PICO format by two independent researchers (34). The extracted data included article details (first author's name and publication year), participant characteristics (number, age, and gender), intervention and comparator details [type of push-up and push-up plus exercises, various unstable surfaces used, location of unstable surfaces (single instability or double instability), and evaluated muscles], and the mean and standard deviation of EMG activity of shoulder primary movers.

### 2.5 Risk of bias assessment

The articles underwent quality assessment using the developed version of Siegfried et al.'s standardized quality assessment tool tailored for observational studies (35), extensively applied in systematic reviews to gauge both external validity, such as a representative sample and participation rate, and internal validity, including performance, detection, selection bias, and control of confounding (28, 29, 36–40). This 11-item tool assigns a total score between 0 (lowest quality) to 11 (highest quality) points.

### 2.6 Data synthesis and statistical analysis

All statistical analyses were performed using Comprehensive Meta-Analysis (CMA) version 3 software at a significance level of *p* < 0.05. The effect size, represented as the standardized mean difference (SMD), was calculated with a 95% confidence interval (CI) to estimate the effects of unstable surfaces on the EMG activity of the AD, PM, and TB muscles during different push-up exercises (41). The interpretation of the SMD was categorized based on Cohen's modified scale: trivial (<0.20), small (0.20–0.59), moderate (0.60–1.19), and large (≥1.20) (42). To examine the potential effect of each study on the overall effect size and assess the robustness of the final results, sensitivity analysis was conducted by excluding studies one by one from the analyses (43). Root mean square (RMS) was computed to quantify the surface EMG amplitude. In studies reporting standard error instead of standard deviation, the following formula was used for the calculation of standard deviation (44): SD = SE*√N (SD = standard deviation, SE = standard error, *N* = sample size).

Heterogeneity among studies was assessed using *Q*-test and *I*^2^ statistic (45), and based on the *I*^2^ statistic, four categories of heterogeneity were considered: low (0%–30%), moderate (31%–50%), high (51%–75%), and very high (76%–100%) (46). Subgroup analysis was performed based on the types of push-up and push-up plus exercises. In all meta-analyses, the random-effects model was used to reduce the potential effect of heterogeneity among studies (47). In addition, publication bias was assessed through the visual interpretation of Begg's funnel plots and Egger's asymmetry test (48, 49). In case of publication bias, the trim and fill method was employed to create a new model and effect size without publication bias (50).

## 3 Results

### 3.1 Literature search

**Figure 1:**
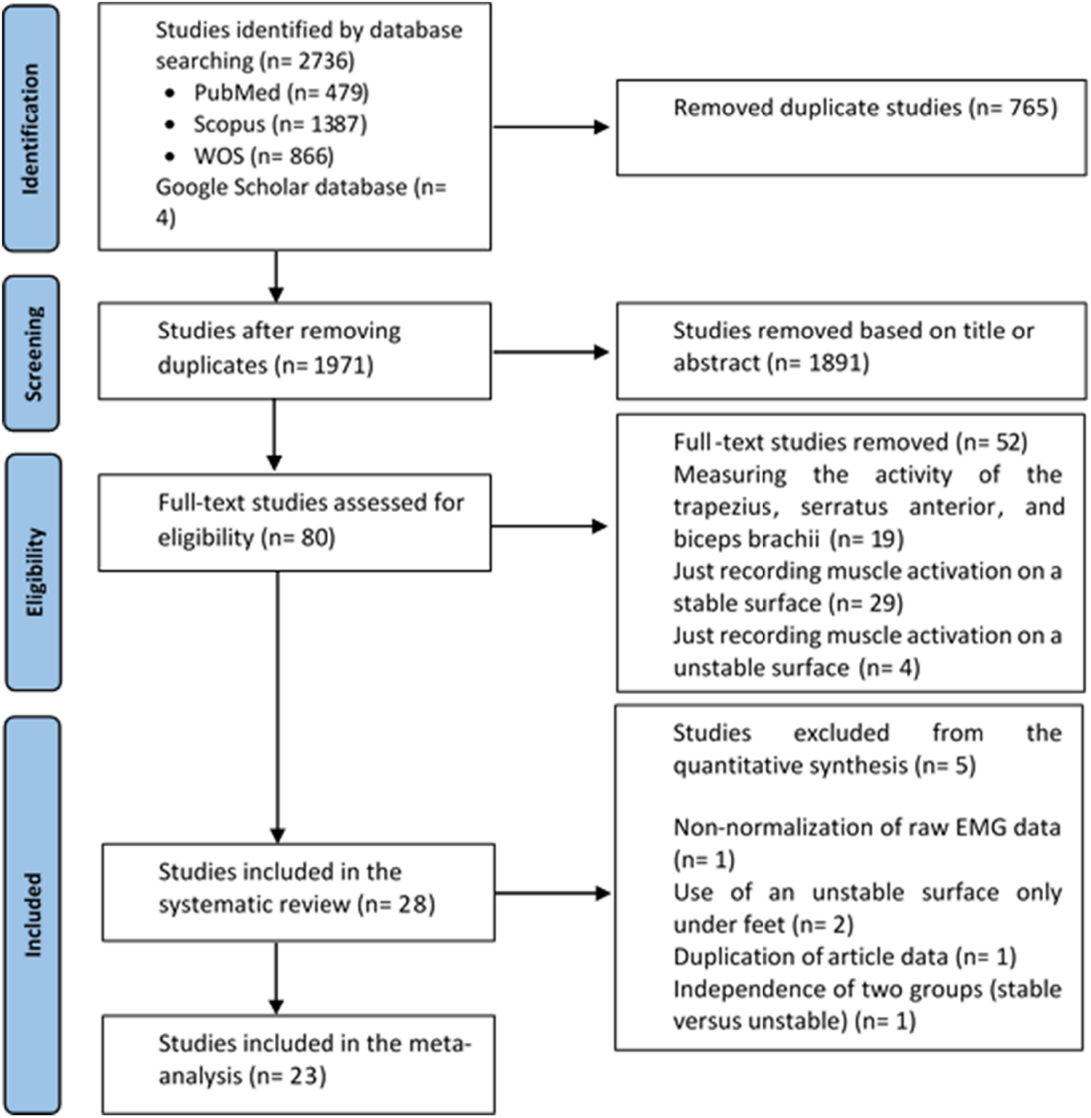
PRISMA flow diagram for the process of searching and identifying studies.

The online search of the databases resulted in the identification of 2,736 articles. After removing duplicate articles, 1,971 studies remained. Following the screening of titles and abstracts, 80 full-text articles were subjected to detailed evaluation. Finally, 28 articles were recognized as eligible studies for inclusion (1, 4, 21, 51–75) (Figure 1). For the meta-analysis, 5 studies out of 28 eligible studies were excluded (1, 56, 60, 68, 72).

### 3.2 Study characteristics

Table 1 presents details of the 28 included studies. All eligible studies were observational, comparing EMG activity of the shoulder primary movers during different types of push-up exercises on stable and unstable surfaces in individuals without scapular dyskinesis. After screening, 652 healthy individuals (136 females, 483 males, and 33 with unspecified gender) were included in the study. Eighteen studies included only male participants (4, 21, 52, 54–57, 59, 60, 62–68, 70, 75), and only one study was conducted solely on female participants (1). Eight studies included both male and female participants (51, 53, 58, 61, 69, 71, 73, 74), while one study did not report the gender of the participants (72). Eight studies included athletes as participants (21, 52, 55, 61, 63, 67, 70, 73), six studies undertook physically active individuals (51, 54, 58, 62, 71, 75), five studies involved students (4, 56, 57, 66, 68), four studies included physically fit individuals (59, 60, 64, 65), one study included both athletes and non-athletes (1), one study included both athletes and physically fit students (74), and one study included conditioning coaches and personal trainers (69). In two studies, the type of participants was not reported (53, 72).

**Table 1:** Characteristics of included studies.

Authors	Participants (male/female)	Age (years)	Intervention(s) (type of PU)	Muscles assessed	Main outcomes
Palma et al. (75)	Healthy (physically active) = 12 (all male)	23.7 ± 3.0	PU on stable and unstable (Bosu ball, balance disc, and sling) [HI]	PM	No differences between stable and unstable surfaces
De Souza Bezerra et al. (52)	Healthy (athletes) = 10 (all male)	26 ± 5	PU on stable and unstable (Swiss ball) [HI, FI]	AD, TB, and PM	AD activity: stable >HI TB activity: HI >FI >stable
Youdas et al. (51)	Healthy (physically active) = 32 (22/10)	Male = 24.6 ± 3.2 Female = 23.6 ± 1.4	PU on stable and unstable (sling and inflated platform) [HI, FI, HFI]	TB, PM, and AD	TB activity: stable <(HI, FI, HFI) and HI >FI
Syed-Abdul et al. (1)	Healthy (athletes and non-athletes) = 69 (all female) Soccer = 24 Gymnasts = 21 Sedentary = 24	18–24	PU on stable and unstable (sling) [HI]	TB and AD	TB activity (soccer): unstable >stable AD activity (soccer and gymnasts): unstable >stable
Harris et al. (53)	Healthy (NR) = 25 (16/9)	27.24 ± 4.02	PU on stable and unstable (sling) [HI]	PM	Unstable >stable
Torres et al. (54)	Healthy (physically active) = 20 (all male)	20.9 ± 1.8	PUP on stable and unstable (Bosu ball) [HFI]	TB, PM, and AD	AD activity: unstable <stable
Phewkham et al. (55)	Healthy (athletes) = 17 (all male)	22.76 ± 2.61	PU on stable and unstable (Swiss ball, sling) [HI]	AD, PM, and TB	AD and PM activities: Swiss ball <stable, and sling TB activity: no differences between the three conditions
Kim et al. (56)	Healthy (students) = 15 (all male)	24.14 ± 0.53	PU on stable and unstable (gym ball and rubber ball) [FI] [feet height = 25, 55 cm]	TB	No differences between stable and unstable surfaces
Lee et al. (57)	Healthy (students) = 20 (all male)	24.05 ± 2.21	Knee PU and Knee PUP on stable and unstable (balance pad) Conditions: 1. feet height = 0 cm (floor) 2. feet height = 25 cm 3. feet height = 30 cm, and HI	PM and TB	TB activity (Knee PU): Condition 2 > Condition 3 > Condition 1
Herrington et al. (58)	Healthy (physically active) = 21 (10/11)	22.8 ± 1.4	Knee PU on stable Standard PU (static) on stable and unstable (Airex pad and Swiss ball) [HI]	AD and PM	Activity of both muscles (stable): Standard PU >Knee PU AD activity: unstable <stable
Borreani et al. (59)	Healthy (physically fit) = 30 (all male)	23 ± 1.13	PU on stable and unstable (wobble board, stability disc, fitness dome, and sling) [HI]	AD	No differences between stable and unstable surfaces
Borreani et al. (60)	Healthy (physically fit) = 29 (all male)	23.5 ± 3.1	PU on stable (hands height = 10 and 65 cm) and unstable (sling: hands height = 10 and 65 cm) [HI]	AD, TB, and PM (clavicular)	TB activity: unstable >stable AD activity: unstable <stable
De Mey et al. (61)	Healthy (athletes) = 47 (26/21)	22 ± 4.31	Half PU and Knee PU on stable and unstable (sling) [HI]	PM and AD	PM activity (Half PU): unstable >stable AD activity (Knee PU): unstable <stable
Maeo et al. (62)	Healthy (physically active) = 20 (all male)	21.4 ± 2.3	PU (static and dynamic) on stable and unstable (sling) [HI]	TB and PM	TB activity (static): unstable >stable TB and PM activities (dynamic): unstable >stable
Calatayud et al. (63)	Healthy (athletes) = 29 (all male)	23.5 ± 3.1	PU on stable (hands height = 10 and 65 cm) and unstable (sling: hands height = 10 and 65 cm) [HI]	AD, TB, and PM (clavicular)	TB activity: unstable >stable
Calatayud et al. (65)	Healthy (physically fit) = 29 (all male)	23.5 ± 3.1	PU on stable and unstable (four types of sling) [HI]	TB, AD, and PM (clavicular)	TB activity: pulley system >all other types AD activity: stable >all other types except the Jungle Gym XT PM activity: Jungle Gym XT >all other types
Calatayud et al. (64)	Healthy (physically fit) = 29 (all male)	22.6 ± 2.6	PU on stable and unstable (two types of sling) [HI]	TB, PM, and AD	TB activity: unstable (pulley system) >stable and unstable (V-shaped system)
McGill et al. (66)	Healthy (students) = 14 (all male)	21.1 ± 2.0	PU on stable and unstable (sling) [HI]	TB, PM, and AD	AD activity: unstable <stable (The significance of the differences between stable and unstable surfaces: NR)
Park et al. (67)	Healthy (athletes) = 16 (all male)	24–26	PU on stable and unstable (wobble board) [HI]	TB	Unstable >stable
Lee et al. (68)	Healthy (students) = 20 (all male) SSG = 10 USG = 10	SSG = 23.3 ± 1.45 USG = 23.7 ± 1.21	Knee PUP on stable and unstable (sling) [HI] Hand position: neutral, internal rotation, and external rotation	PM	No differences between stable and unstable surfaces
Anderson et al. (69)	Healthy (conditioning coaches and personal trainers) = 15 (10/5)	29.3 ± 6.4	PU on stable and unstable (stability ball and balance board) [HI, FI, HFI]	TB	Unstable (HFI) >FI >HI >stable
Park and Yoo (70)	Healthy (athletes) = 14 (all male)	22 ± 2	PU on stable and unstable (wobble board) [HI]	TB and PM	PM activity: unstable >stable TB and PM activities: up phase >down phase
Snarr and Esco (71)	Healthy (physically active) = 21 (15/6)	Male = 25.93 ± 3.67 Female = 23.5 ± 1.97	PU on stable and unstable (sling) [HI]	AD, TB, and PM	activity of all muscles: unstable >stable
Kim et al. (72)	Healthy (NR) = 33 (NR)	21.61 ± 1.32	PU on stable and unstable (exercise ball) [FI] Conditions (feet height = 65 cm): 1. Foot table 2. Knee table 3. Foot ball 4. Knee ball	PM and TB	PM activity: foot ball >knee ball, foot ball >knee table
Sandhu et al. (4)	Healthy (students) = 30 (all male)	20–30	Standard PU, Knee PU, Elbow PU, and Wall PU on stable and unstable (Swiss ball) [HI]	TB and PM	PM activity [standard PU (up and down phases) and Wall PU (down phase)]: unstable >stable TB and PM activities [Elbow PU (down phase)]: unstable >stable
Marshall and Murphy (73)	Healthy (athletes) = 12 (8/4)	22.1 ± 2.4	PU on stable and unstable (Swiss ball) [HI]	TB and PM	TB activity (up and down phases): unstable >stable
Lehman et al. (21)	Healthy (athletes) = 13 (all male)	26.3 ± 1.5	PU and PUP on stable and unstable (Swiss ball) [HI, FI]	TB and PM	TB activity (PU and PUP): unstable (only the HI) >stable
Freeman et al. (74)	Healthy (athletes and physically fit students) = 10 (9/1)	22–34	PU on stable and unstable (basketball ball) [HI]	TB, PM, and AD	PM activity: unstable >stable

NR, not reported; SSG, stable surface group; USG, unstable surface group; PU, push-up; PUP, push-up plus; HI, hand instability; FI, feet instability; HFI, hand and feet instability; PM, pectoralis major; TB, triceps brachii; AD, anterior deltoid.

The identified common push-up exercises were standard push-up, knee push-up, elbow push-up, wall push-up, half push-up, push-up plus, and knee push-up plus. Various unstable surfaces were used, such as stability balls, exercise balls, basketball balls, Swiss balls, Bosu^TM^ balls, gym balls, rubber balls, slings, inflated platforms, balance pads, Airex^TM^ pads, wobble boards, balance boards, stability (balance) discs, and fitness domes. Among these, 25 studies evaluated unstable surfaces under the hands (1, 4, 21, 51–53, 55, 57–71, 73–75), six studies evaluated unstable surfaces under the feet (21, 51, 52, 56, 69, 72), and three studies evaluated both hands and feet (double instability) (51, 54, 69).

Regarding the normalization methods for EMG signals, maximum voluntary isometric contraction (MVIC) was used in 15 studies (4, 51, 52, 56–61, 63–65, 68, 73, 75), maximal voluntary contraction (MVC) in eight studies (21, 55, 62, 66, 67, 70, 71, 74), reference voluntary isometric contraction (RVIC) in one study (54), reference voluntary contraction (RVC) in one study (72), and reference isometric contraction (RIC) in one study (53). One study did not report the normalization method used (69), and in one study, the signals were not normalized (1). It is also important to note that in all studies except one, EMG activity of the primary shoulder movers was analyzed using RMS (1).

### 3.3 Risk of bias assessment

Appendix 1 displays a comprehensive assessment of the included studies. Only eight studies included both male and female participants (51, 53, 58, 61, 69, 71, 73, 74), which may decrease external validity. All participants in the 28 selected studies completed the exercises fully without dropouts. However, none of the studies blinded the assessors during EMG activity measurements, which is a risk factor for increased bias. Due to the observational nature of EMG analysis, blinding of assessors was not possible. Only one study included physical examination to assess scapular dyskinesis or ensure normal scapulohumeral rhythm (51), potentially affecting internal validity. Two studies did not randomize the order of exercises, which could introduce a potential fatigue-related selection bias (58, 69). Nine studies did not include a familiarization session for participants to become acquainted with the various push-up exercises and their proper execution (21, 52, 53, 55–58, 67, 72). Additionally, 12 studies did not standardize the push-up exercise using participant height (for hand and foot placement) or a metronome for controlling movement speed (1, 4, 21, 52, 53, 56–58, 68, 72, 73, 75). Except for one study, appropriate normalization of raw EMG data was performed in all studies (1). However, only two studies randomized the order of MVICs (53, 74), which might affect the internal validity of the results. The quality of the studies was generally good, as indicated by the average quality score of 6.46, which varied from 4 to 9 points.

### 3.4 Qualitative analysis

The push-up exercises used in the final studies were standard push-up, knee push-up, elbow push-up, wall push-up, half push-up, push-up plus, and knee push-up plus. The muscles under investigation included the AD (15 studies) (1, 51, 52, 54, 55, 58–61, 63–66, 71, 74), PM (23 studies) (4, 21, 51–55, 57, 58, 60–66, 68, 70–75), and TB (22 studies) (1, 4, 21, 51, 52, 54–57, 60, 62–67, 69–74). Additionally, the positive effect of using unstable surfaces during various types of push-up on AD muscle activity was reported in 2 studies (1, 71), on PM muscle in 7 studies (4, 53, 61, 62, 70, 71, 74), and on TB muscle in 14 studies (1, 4, 21, 51, 52, 57, 60, 62, 63, 65, 67, 69, 71, 73).

### 3.5 Meta-analysis outcomes

**Figure 2:**
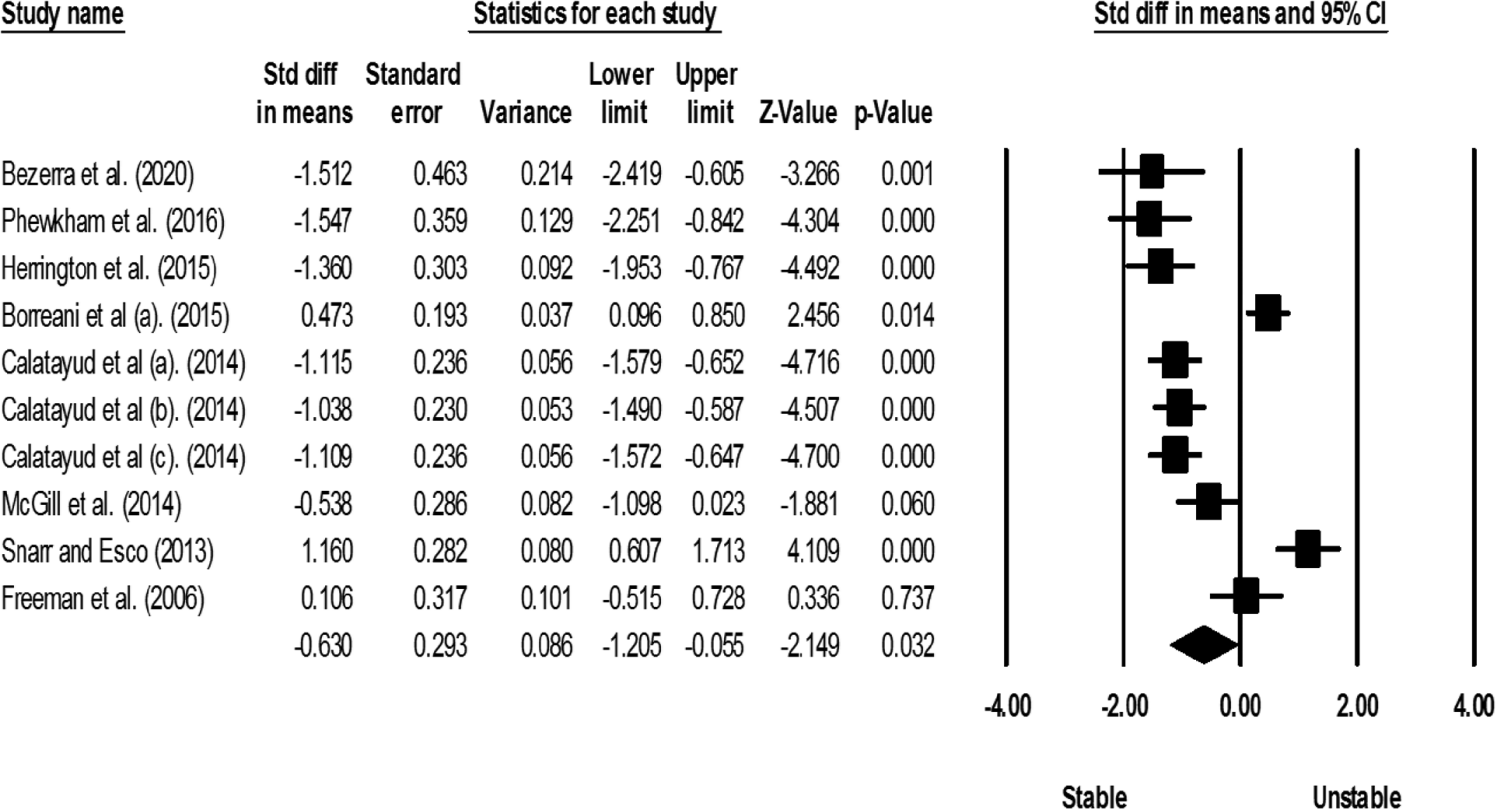
Forest plot of the meta-analysis of the effect of an unstable surface on the AD muscle EMG activity during push-up.

**Figure 3:**
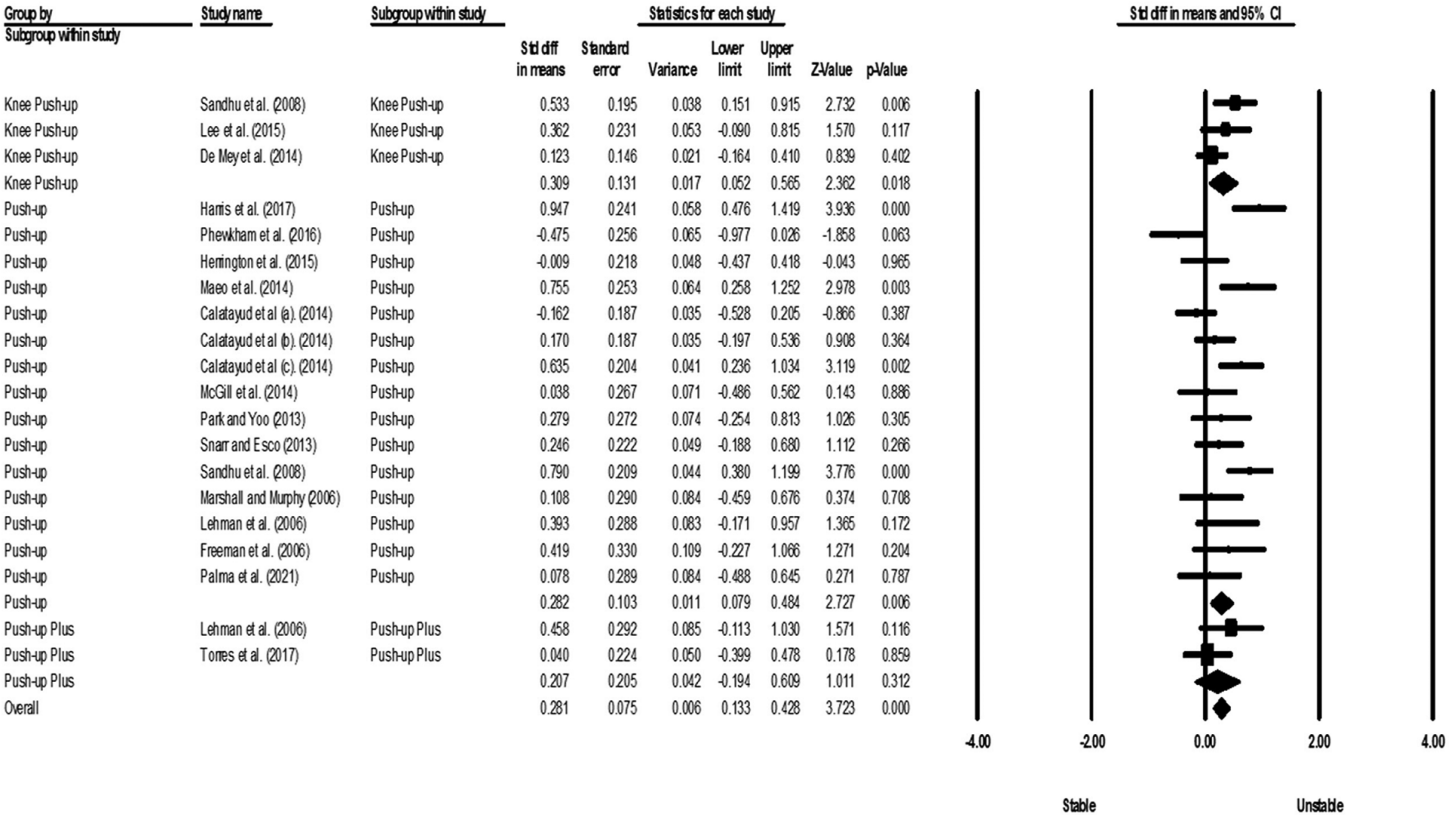
Forest plot of the meta-analysis of the effect of an unstable surface on the PM muscle EMG activity during various types of push-up.

**Figure 4:**
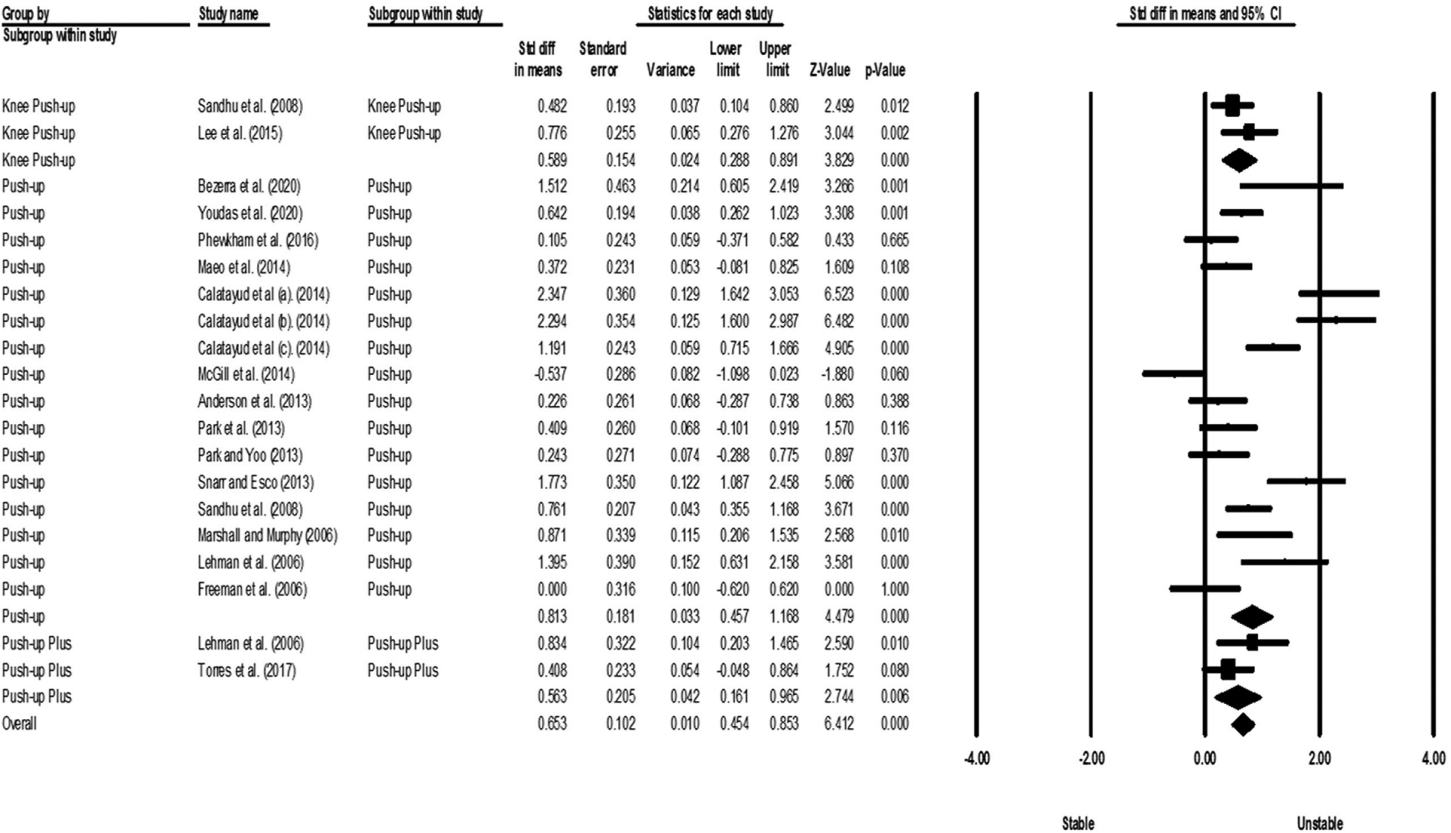
Forest plot of the meta-analysis of the effect of an unstable surface on the TB muscle EMG activity during various types of push-up.

Studies were grouped according to exercises and muscles to ascertain the effect of unstable surfaces on the activity of shoulder primary mover muscles. The results for the AD, PM, and TB muscle activations in various types of push-up are shown in Figures 2–4. Figure 2 illustrates the results of AD muscle activity during push-up. The meta-analysis results showed that adding unstable surfaces reduces AD muscle activity during push-up [*P* = 0.032; *I*^2^ = 91.34%; SMD = −0.630 (95% CI −1.205, −0.055)]. Figure 3 illustrates the results of PM muscle activity during different types of push-up. Subgroup analysis showed no significant difference in PM muscle activity between stable and unstable surfaces during push-up plus [*P* = 0.312; *I*^2^ = 22.82%; SMD = 0.207 (95% CI −0.194, 0.609)]. However, using unstable surfaces increased PM muscle activity during knee push-up [*P* = 0.018; *I*^2^ = 32.29%; SMD = 0.309 (95% CI 0.052, 0.565)] and push-up [*P* = 0.006; *I*^2^ = 63.72%; SMD = 0.282 (95% CI 0.079, 0.484)]. Figure 4 shows the results of TB muscle activity during different types of push-up. Subgroup analysis indicated an increase in TB muscle activity with the application of unstable surfaces during knee push-up [*P* = 0.000; *I*^2^ = 0.00%; SMD = 0.589 (95% CI 0.288, 0.891)], push-up [*P* = 0.000; *I*^2^ = 85.05%; SMD = 0.813 (95% CI 0.457, 1.168)], and push-up plus [*P* = 0.006; *I*^2^ = 13.16%; SMD = 0.563 (95% CI 0.161, 0.965)].

**Figure 5:**
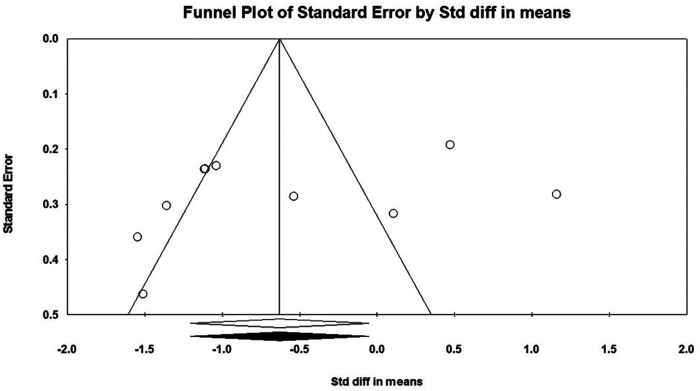
Begg's funnel plot of the AD muscle EMG activity with 95% confidence interval (push-up).

**Figure 6:**
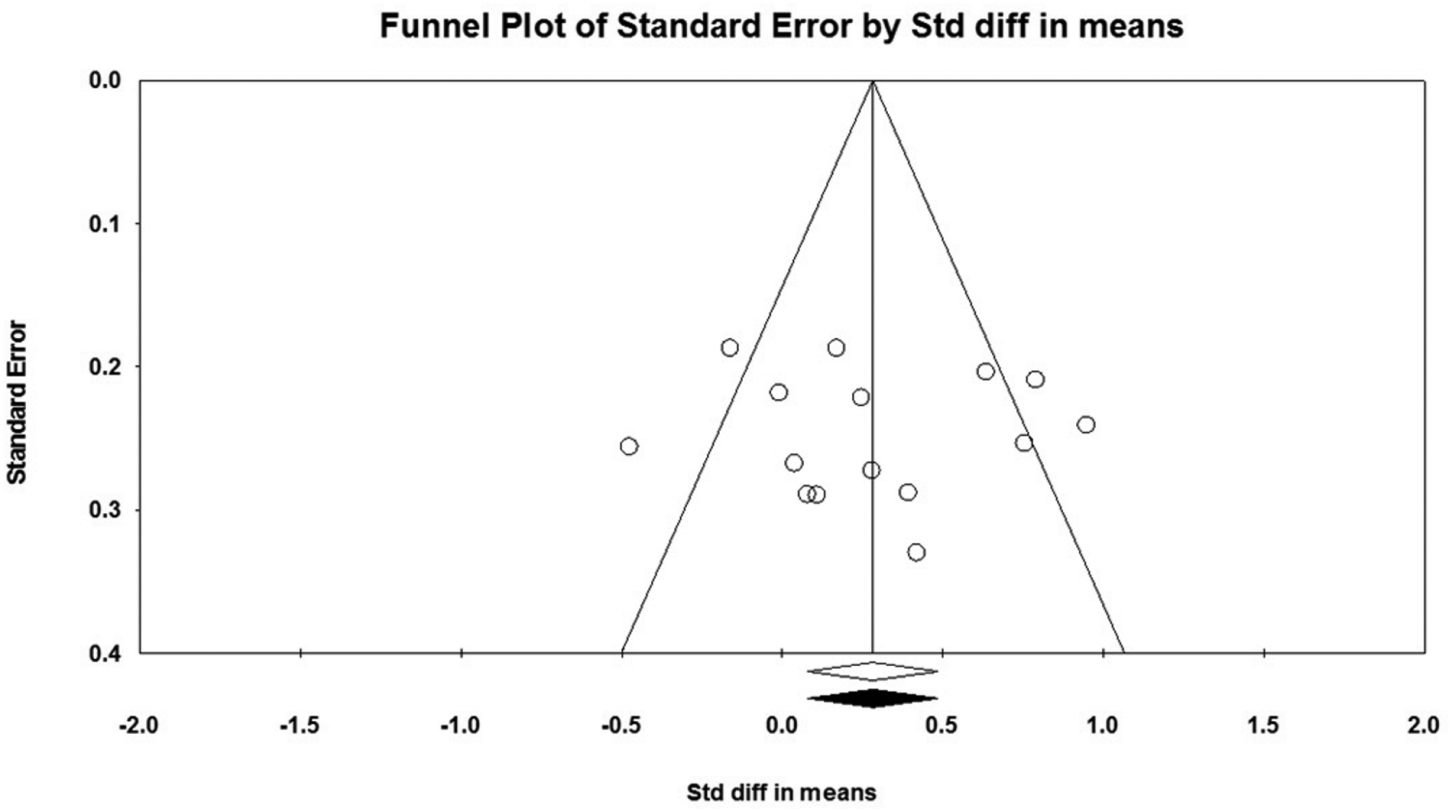
Begg's funnel plot of the PM muscle EMG activity with 95% confidence interval (push-up).

**Figure 7:**
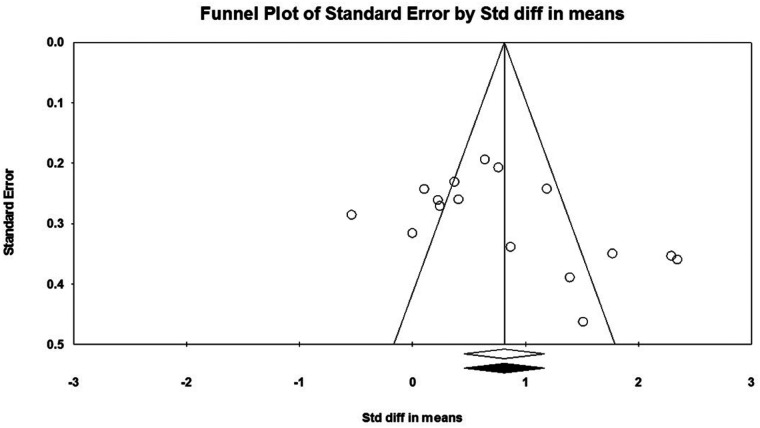
Begg's funnel plot of the TB muscle EMG activity with 95% confidence interval (push-up).

**Figure 8:**
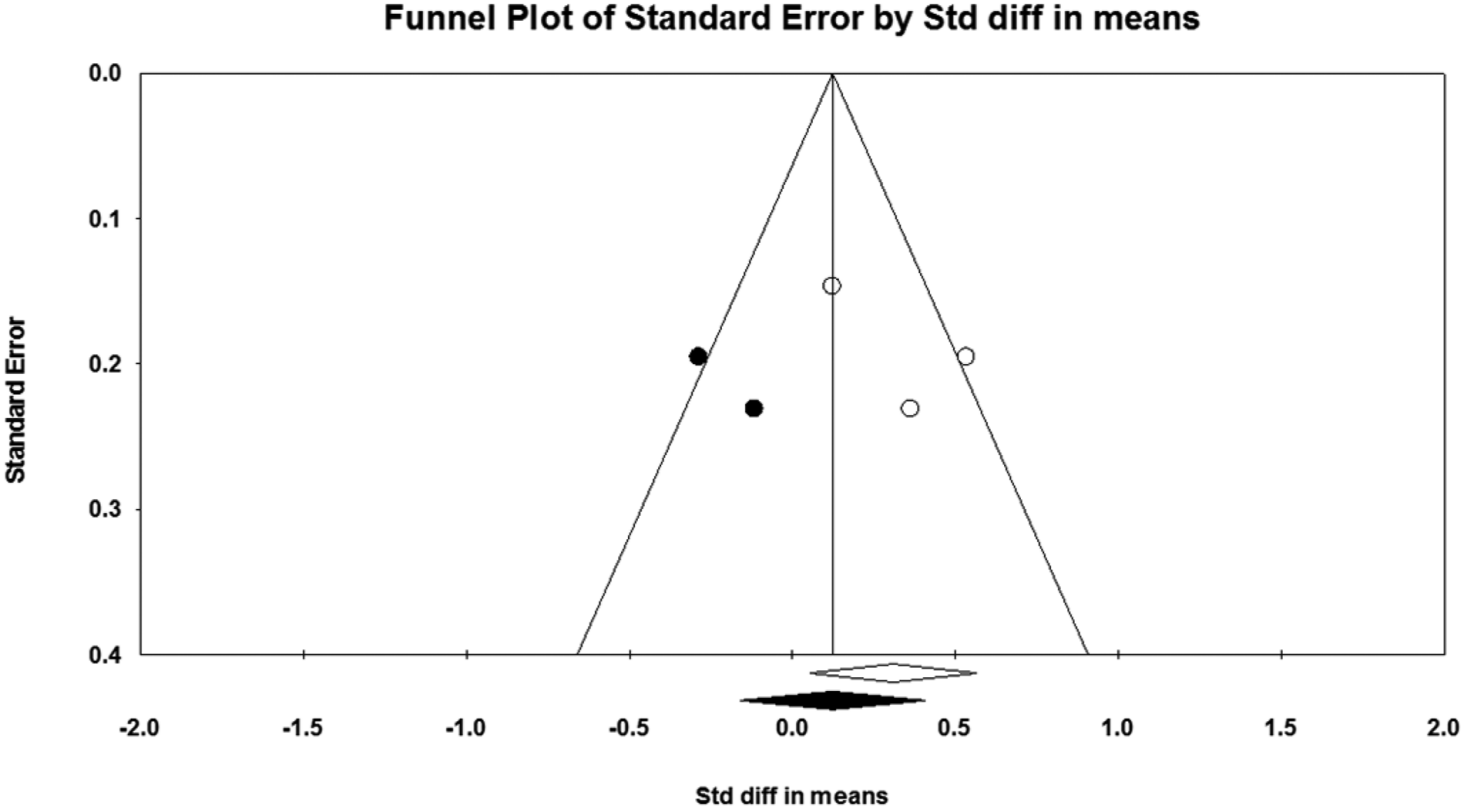
Begg's funnel plot of the PM muscle EMG activity with 95% confidence interval (knee push-up).

Considering that there must be at least three studies to evaluate the publication bias, it was not possible to evaluate the publication bias for PM in push-up plus studies and TB in push-up plus and knee push-up studies due to the small number of studies (2 studies). In addition, no evidence of publication bias was observed through visual evaluation of Begg's funnel plots and Egger's test results for AD (*P* = 0.362), PM (*P* = 0.927), and TB (*P* = 0.073) muscles during push-up (Figures 5–7). In the meta-analysis of PM, the absence of publication bias was confirmed using Egger's test for knee push-up (*P* = 0.448) studies. However, due to the uneven distribution of studies in Begg's funnel plot, the trim and fill method was performed based on the random-effects model. Trim and fill analysis for the PM in knee push-up (2 imputed studies) led to a significant change in the final result (Figure 8).

### 3.6 Sensitivity analysis

**Figure 9:**
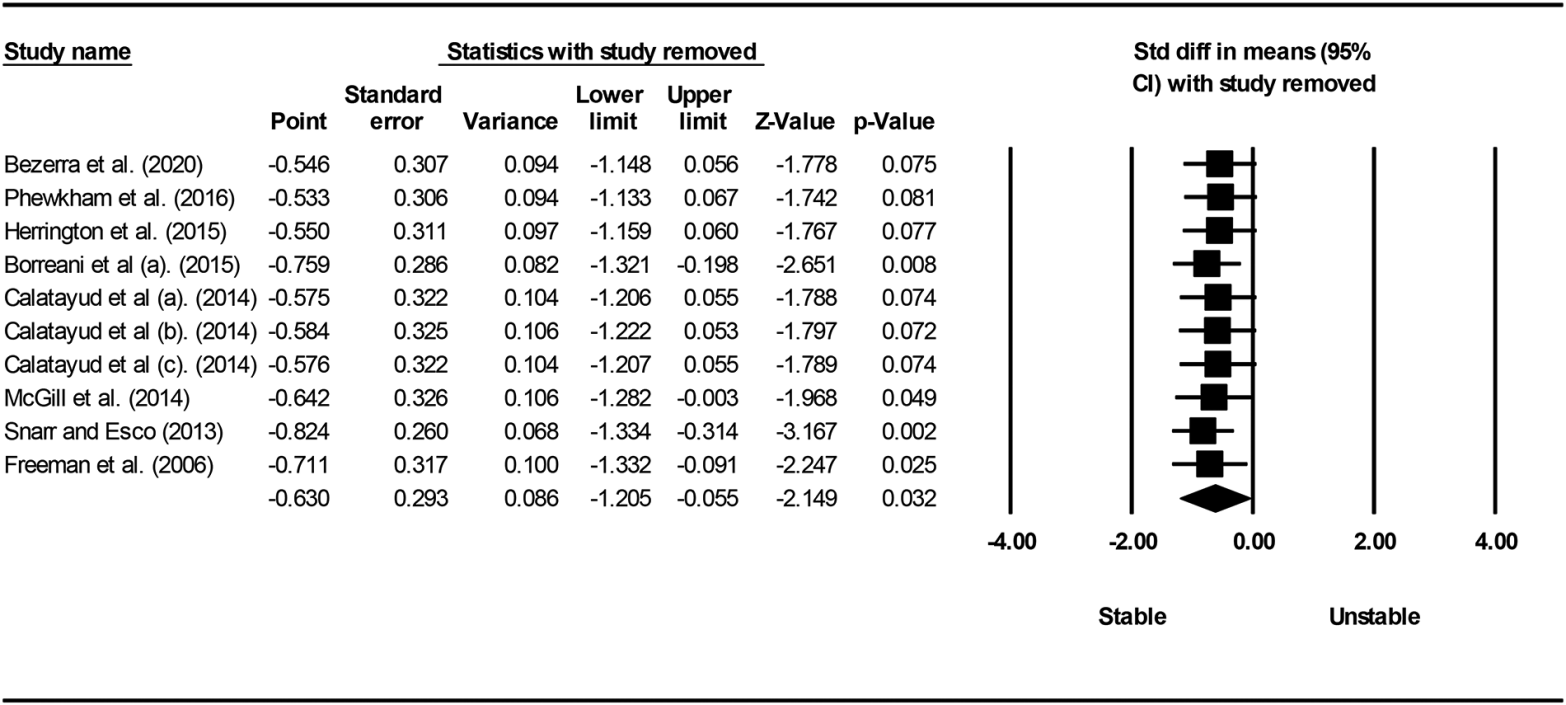
Forest plot of sensitivity analysis with one-by-one exclusion of studies from the meta-analysis of AD muscle during push-up.

**Figure 10:**
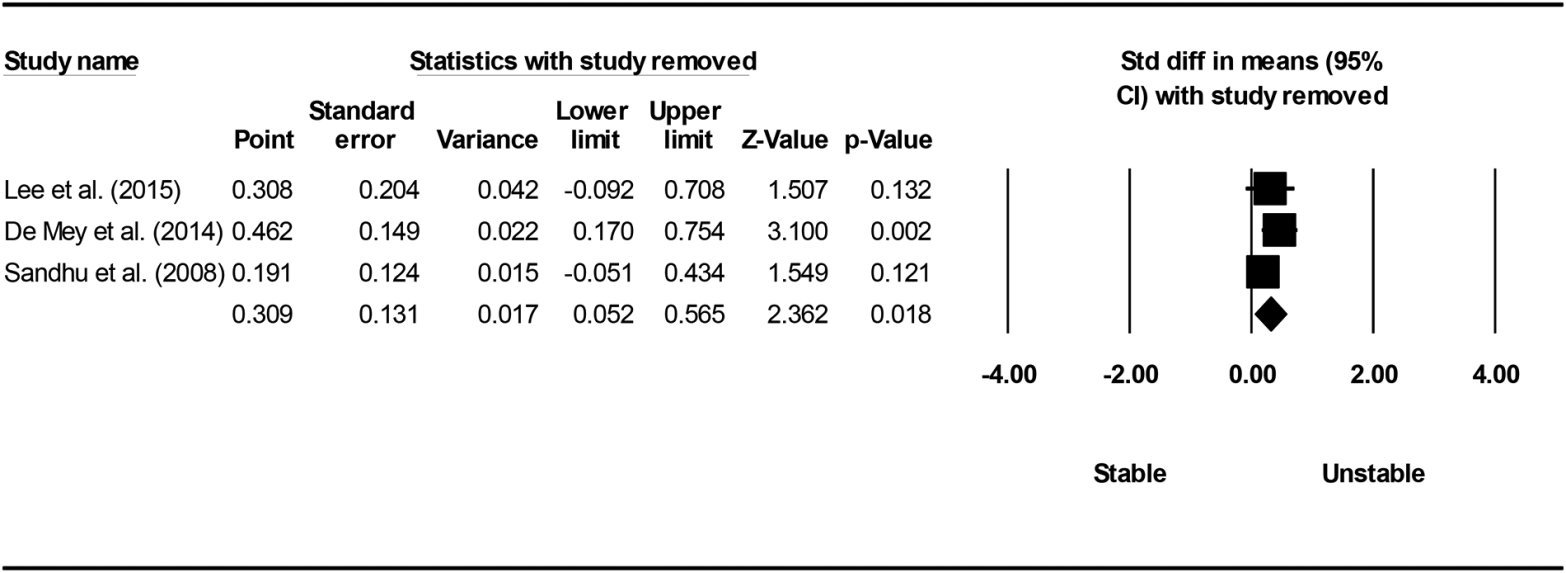
Forest plot of sensitivity analysis with one-by-one exclusion of studies from the meta-analysis of PM muscle during knee push-up.

Sensitivity analysis showed that excluding studies one by one did not affect the meta-analysis results for the PM muscle during push-up and push-up plus, as well as for the TB muscle during knee push-up and push-up, indicating the robustness of the analyses. Other sensitivity analyses showed that excluding each of the studies by De Souza Bezerra et al. (52), Phewkham et al. (55), Herrington et al. (58), Calatayud et al. (63), Calatayud et al. (65), and Calatayud et al. (64) from the meta-analysis of the AD muscle during push-up and Sandhu et al. (4) and Lee et al. (57) from the meta-analysis of the PM muscle during knee push-up significantly changed the results. In other words, despite the meta-analysis findings indicating a decrease in AD muscle activity during push-up and an increase in PM muscle activity during knee push-up when unstable surfaces were introduced, sensitivity analysis demonstrated that the utilization of unstable surfaces did not significantly affect the activity of the AD during push-up (Figure 9) nor the PM during knee push-up (Figure 10).

## 4 Discussion

The aim of the current research was to identify common types of push-up exercises and determine the effects of unstable surfaces on the EMG activity of shoulder primary mover muscles (AD, PM, and TB) in individuals without scapular dyskinesis during the performance of these exercises. The results demonstrated that unstable surfaces can modulate muscular activity depending on the biomechanical affordances of the exercise, indicating that different muscles are stimulated to varying degrees. Therefore, the discussion should delve into the specific details of each muscle to better understand the potential reasons for different behaviors when facing unstable surfaces during various push-up exercises.

### 4.1 AD muscle

The meta-analysis results revealed that the use of unstable surfaces during push-up exercises in individuals without scapular dyskinesis led to a decrease in EMG activity of the AD muscle (SMD = 0.630). This finding aligns with previous literature, which also suggests that the amplitude of EMG activity of the AD is generally greater for stable surfaces than unstable ones (52, 54, 55, 58, 60, 61, 64, 66). Despite the shoulder girdle muscles acting as stabilizers during push-up, the AD may be considered as an agonist muscle during this specific movement (52, 76).

Furthermore, the type of push-up exercise and hand positioning during its execution are crucial factors that may influence the results. For instance, De Mey et al. (61) demonstrated decreased AD activity during knee push-up on a sling compared to a stable surface. Conversely, increased AD activity was observed during push-up on a sling in the same study. The increase in activity can be attributed to hand placement during exercise. In this study, the hands were positioned slightly wider than the shoulder width, placing the AD under its greatest length and tension, especially when combined with horizontal abduction (concentric phase) (61). Therefore, clinicians and coaches should consider this aspect when aiming to increase AD involvement, as stable surfaces seem to offer the greatest benefits. However, it is essential to acknowledge that Syed-Abdul et al. (1) and Snarr and Esco (71) reported increased AD activity during unstable surface usage (1, 71). This increase could be related to gender and participant characteristics, as the study by Syed-Abdul et al. (1) included female athletes (soccer players and gymnasts), and Snarr and Esco (71) included both active males and females with at least 6 months of resistance training experience.

Additionally, it is worth mentioning that EMG signals were not normalized in the study by Syed-Abdul et al. (1), and muscular activity was expressed as an absolute integral. This could have influenced the findings. On the other hand, Youdas et al. (51) and Freeman et al. (74) reported no changes in AD activity during stable and unstable push-up (51, 74). The result in the study by Youdas et al. (51) is possibly related to participant experience, as participants were instructed to perform stable and unstable push-up for at least 2 weeks before data collection to prevent suboptimal performance due to inexperience with the suspension training system (51). The absence of changes in AD activity in the Freeman et al. (74) study could be attributed to the specific unstable surface used (basketballs). These basketballs may not have provided a sufficient level of instability to induce changes in muscular activity (74).

Although our meta-analysis results indicated a moderate effect of using unstable surfaces on the activity of the AD muscle, there was very high heterogeneity in the studies. This could be attributed to the different unstable surfaces (Swiss balls, basketball balls, wobble boards, and slings) used in the studies. Sensitivity analysis also confirmed that the overall result of the AD muscle during push-up is influenced by studies utilizing Swiss balls and slings as unstable surfaces (52, 55, 58, 63–65).

### 4.2 PM muscle

The meta-analysis results demonstrated that the use of unstable surfaces during push-up (SMD = 0.282) and knee push-up (SMD = 0.309) in individuals without scapular dyskinesis led to an increase in the EMG activity of the PM muscle. However, the unstable surface did not have a significant effect on the EMG activity of the PM during push-up plus exercises. Although most evidence indicates higher EMG activity of the PM during push-up on unstable surfaces (4, 53, 61, 62, 64, 70, 71, 74), it is crucial to consider the type of unstable surface used and the phase of the exercise, as they can influence the level of muscle activation.

In recent years, the use of slings as an unstable surface has gained popularity among researchers. Slings can create instability in multiple directions since there is no contact with the ground. Thus, the increased PM activity during unstable surface usage might be due to the need for shoulder abduction control when using the sling (61). The PM is responsible for horizontal and oblique adduction, accompanied by internal rotation of the arm (71). Some authors who have used exercise balls as an unstable surface have not observed any differences in PM activation (21, 52, 54, 58, 73, 75) or even reported decreased activation of the PM (55). Considering the phase of the exercise, Park and Yoo (70) demonstrated that shoulder adduction and scapular protraction resulting from concentric contraction of the PM lifted the body mass during the up phase of the push-up exercise (70). Thus, it seems that an unstable support surface might demand more PM activity during the up phase of the push-up. However, Sandhu et al. (4) observed a significant increase in PM activity during the eccentric phase (4). The conflicting results of these two studies can be attributed to the type of push-up exercise and the type of unstable surface used. Park and Yoo (70) used standard push-up and a wobble board, while Sandhu et al. (4) used elbow push-up and a Swiss ball.

Visual assessment of the funnel plot for the PM muscle during push-up showed no evidence of publication bias. However, there is high heterogeneity among studies. Possible sources of this high heterogeneity include the gender of participants, different unstable surfaces used, and various methods of EMG signal normalization. Nevertheless, sensitivity analysis did not show any noticeable changes after removing studies one by one from the meta-analysis of the PM muscle during push-up. Further, although the heterogeneity among studies examining the effect of unstable surfaces on the activity of the PM muscle during knee push-up was moderate, sensitivity analysis indicated that the overall findings are likely dependent on the gender and level of physical activity of the participants in studies by Lee et al. (57) and Sandhu et al. (4). These two studies consisted of male students, whereas the study by De Mey et al. (61) included both male and female athletes.

In justifying the lack of a significant effect of an unstable surface on the EMG activity of the PM during push-up plus exercises, it is necessary to note that the degree of instability applied during the exercise can influence muscle activation (54). According to the review study by Behm and Colado (24), applying excessive instability during the execution of an exercise can lead to reduced activation of agonist muscles (24). Thus, in the study by Torres et al. (54), where double instability (hands and feet) was employed, the excessive level of instability might be a potential reason for the lack of effect on PM activity. In fact, simultaneous use of unstable surfaces for hands and feet may increase the difficulty level or task demands, leading to increased activation of scapular stabilizer muscles and decreased activation of glenohumeral muscles (agonists) (54). Additionally, Lehman et al. (21) suggested that the primary focus of the PM might be on its main movement, and it may have a lesser role in responding to stability changes (21). In other words, stability-related changes might be controlled by other muscles influencing the shoulder joint. These findings support the recent hypothesis proposed by Youdas et al. (51), which focuses on the “amount of load lifted” and suggests that although muscle recruitment might increase in the primary movers during suspended and unstable conditions, it might not change when the lifted load (body weight) is kept constant (51).

### 4.3 TB muscle

The results of the meta-analysis showed that using unstable surfaces during push-up (SMD = 0.813), push-up plus (SMD = 0.563), and knee push-up (SMD = 0.589) exercises in individuals without scapular dyskinesis led to an increase in the activity of the TB muscle. Generally, studies comparing the activity of the TB muscle during different types of push-up exercises on stable and unstable surfaces have reported either increased activity (1, 4, 21, 51, 52, 60, 62–65, 67, 69, 71, 73) or no difference (1, 21, 54–56, 70, 72, 74) when adding an unstable surface.

The significant increase in TB muscle activity on the unstable surface may be due to its biomechanical function. The TB muscle is considered the main elbow extensor, assisted by the anconeus, and it is a biarticular muscle since its long head attaches to the infraglenoid tubercle of the scapular, making it also an important shoulder extensor (77, 78). Thus, it requires stability and mobility since it is involved in both elbow and shoulder movements (4, 21).

Reports from studies using unstable tools indicate that performing exercises under unstable conditions increases the activity of limb muscles to control limb position and perform precise movements (24, 79–81). The current findings support this theory and suggest that the increased activity of upper limb muscles is a specificity of push-up exercises based on a sling in the studies by Youdas et al. (51), Syed-Abdul et al. (1), Borreani et al. (60), Maeo et al. (62), Calatayud et al. (63), Calatayud et al. (64), and Snarr and Esco (71), where unstable grips must be maintained by elbow flexor and extensor muscles in their respective positions.

Regarding push-up exercise, attention to the phase of the exercise is essential. Park and Yoo (70) demonstrated that the TB muscle is more active during the concentric phase (up phase) of push-up exercise than during the eccentric phase (down phase) (70). This is consistent with the force-EMG relationship, indicating that eccentric contractions use more elastic components and metabolic processes more effectively than concentric contractions. Consequently, for an equal level of muscular tension, an eccentric contraction requires fewer motor units (lower EMG activity) compared to a concentric contraction (82).

Furthermore, during the concentric phase of the push-up exercise, as the shoulder flexes, the elbow starts to extend, meaning that the long head of the TB muscle is isometrically contracted to a great extent, while the medial and lateral heads are concentrically contracted (51). During the eccentric phase, the TB muscle is elongated. Performing intense eccentric exercises may cause microstructural damage to the muscles (e.g., delayed onset muscle soreness) (83). Krentz and Farthing (84) stated that performing intense eccentric exercises within 48 h could lead to long-term muscle weakness (84).

Given the potential risks associated with the eccentric phase of the push-up on an unstable surface, it may not offer any advantage, and it could even pose a risk of injury. Therefore, push-up plus exercise is a better alternative, especially for non-athletes.

On the other hand, Sandhu et al. (4) observed a significant increase in the activity of the TB muscle during the eccentric phase of push-up exercise (4). The different types of push-up and unstable surfaces used in Park and Yoo (70) and Sandhu et al. (4) studies might explain these conflicting results. Park and Yoo (70) used a standard push-up and a wobble board, while Sandhu et al. (4) used an elbow push-up and a Swiss ball in their research.

Our findings for the TB muscle, based on effect size, indicate a progressive increase in neuromuscular demand with various types of push-up on unstable surfaces. These findings can be applied to progressive prescription of shoulder muscle exercises in different training phases and potentially in the rehabilitation of individuals with shoulder musculoskeletal disorders.

Although most studies have reported higher EMG activity in the TB muscle during various push-up exercises on an unstable surface, some researchers have not found a significant difference between stable and unstable surfaces (1, 21, 54–56, 70, 72, 74). These differing results can be justified based on factors such as sex, EMG signal normalization methods, location of the unstable surface, and type of unstable surface used. The study by Syed-Abdul et al. (1) was the only one that included female participants (gymnasts and sedentary women) and did not normalize the EMG signals. The location of the unstable surface was under the hands and feet (double instability) in the study of Torres et al. (54), while in the studies by Kim et al. (56), Kim et al. (72), and Lehman et al. (21), it was only under the feet. In contrast, in studies that reported increased activity, the unstable surface was only under the hands. The behavior of the TB muscle observed by Torres et al. (54) might be related to the applied double instability. In three other studies, the proximal part of the body must be controlled to ensure steady movement in the distal parts of the body. The free movement of the distal part through increased stability in the proximal part is known as “feedforward control” (72). In these studies, the stabilizers of the shoulder complex might also be contracted under unstable conditions to ensure the stability of the unstable distal parts. This argument is consistent with the results of Naughton et al. (85), who found that the muscular activity of the proximal parts is necessary to perform movements in the distal parts (85).

However, considering the same height of the ball and bench in these three studies, gravitational force remained constant on the hands, and the activity of the TB muscle did not significantly increase. Moreover, Torres et al. (54) and Kim et al. (72), unlike other studies that normalized the signals based on MVIC or MVC, used the RVIC and RVC, respectively, for signal normalization in their raw EMG data. Freeman et al. (74) also demonstrated that performing a push-up with two hands on two basketballs and on a stable surface resulted in the same level of TB muscle activity. The same activity of the TB muscle on both stable and unstable surfaces may be due to the insufficient degree of instability in basketballs.

## 5 Limitations

The majority of the included studies focused on healthy male individuals, which may limit the generalizability of the findings to females and males with shoulder injuries. Additionally, all the studies in this review assessed muscular activity in non-fatigued states. Given that muscular activity levels can change with fatigue (86), and considering that addressing fatigue and improving endurance capacity are key priorities in prevention and rehabilitation programs for returning to sports, it is advisable to exercise caution when applying these results to exercises performed in a fatigued state. The focus solely on various types of push-up and the neglect of assessing other closed kinetic chain shoulder exercises, pooling different unstable surfaces into a single database without considering potential differences in neuromuscular demand for each unstable surface, as well as the low methodological quality of some included studies, are other limitations of our study.

## 6 Suggestions for further research

Considering that rotator cuff muscles and trunk stabilizer muscles play a crucial role in upper body prevention or rehabilitation programs, it is recommended that future researchers to investigate the EMG activity of these muscle groups during various types of push-up and push-up plus exercises. Additionally, future research should focus on examining motor control variables such as timing and muscle activation patterns of the shoulder girdle during different types of push-up and push-up plus. Finally, it is suggested that future research should explore the kinematics of movement during the execution of various push-up and push-up plus variations to facilitate a better understanding of the relationships between the studied muscle activity and the scapulohumeral rhythm.

## 7 Implications for practice

Our findings provide valuable insights for designing shoulder girdle injury prevention strategies. It is evident that the choice of support surface (stable or unstable) and the type of push-up or push-up plus significantly impact the EMG activity of key shoulder muscles. In particular, the most suitable exercise for increasing AD muscle activity is push-up on a stable surface. To concurrently increase activity of the PM and TB muscles, adding unstable surfaces under hands during knee push-up and standard push-up is recommended.

## 8 Conclusions

Unstable surfaces can have varying effects on muscle activity depending on the biomechanical nature of the exercise. This means that using an unstable surface does not necessarily lead to an increase in the activity of all the shoulder muscles. The results of this review provide a framework for coaches, practitioners, and sports therapists to choose the most suitable type of exercise for designing training or rehabilitation programs. Prescribing various types of push-up gradually increases muscle activity and prepares individuals for upper limb control and safe return to sports by addressing strength needs. Therefore, when selecting exercises, coaches and sports therapists should consider the desired muscle activation patterns and choose the appropriate exercises accordingly. The use of unstable surfaces can have different effects on muscle activity, and these effects depend on the specific muscle targeted and the type of exercise being performed.

## Data Availability

The original contributions presented in the study are included in the article/Supplementary Material, further inquiries can be directed to the corresponding author.

## Appendix

**Table:** Appendix 1 Quality assessment of included studies.

Study	External validity		Internal validity									Score
			Performance	Detection		Selection bias and control of confounding						
Authors	Representative sample	Participation rate	Direct observation	Blind rater	Physical examination	Randomization of exercises	Familiarization of exercises	Standardization of exercise technique	Randomization of MVIC_s_	Appropriate normalization	Appropriate statistical tests	Total score
Palma et al. (75)	No	Yes	Yes	No	No	Yes	Yes	No	No	Yes	Yes	6
De Souza Bezerra et al. (52)	No	Yes	Yes	No	No	Yes	No	No	No	Yes	Yes	5
Youdas et al. (51)	Yes	Yes	Yes	No	Yes	Yes	Yes	Yes	No	Yes	Yes	9
Syed-Abdul et al. (1)	No	Yes	Yes	No	No	Yes	Yes	No	No	No	Yes	5
Harris et al. (53)	Yes	Yes	Yes	No	No	Yes	No	No	Yes	Yes	Yes	7
Torres et al. (54)	No	Yes	Yes	No	No	Yes	Yes	Yes	No	Yes	Yes	7
Phewkham et al. (55)	No	Yes	Yes	No	No	Yes	No	Yes	No	Yes	Yes	6
Kim et al. (56)	No	Yes	Yes	No	No	Yes	No	No	No	Yes	No	4
Lee et al. (57)	No	Yes	Yes	No	No	Yes	No	No	No	Yes	No	4
Herrington et al. (58)	Yes	Yes	Yes	No	No	No	No	No	No	Yes	Yes	5
Borreani et al. (59)	No	Yes	Yes	No	No	Yes	Yes	Yes	No	Yes	Yes	7
Borreani et al. (60)	No	Yes	Yes	No	No	Yes	Yes	Yes	No	Yes	Yes	7
De Mey et al. (61)	Yes	Yes	Yes	No	No	Yes	Yes	Yes	No	Yes	Yes	8
Maeo et al. (62)	No	Yes	Yes	No	No	Yes	Yes	Yes	No	Yes	Yes	7
Calatayud et al. (63)	No	Yes	Yes	No	No	Yes	Yes	Yes	No	Yes	Yes	7
Calatayud et al. (65)	No	Yes	Yes	No	No	Yes	Yes	Yes	No	Yes	Yes	7
Calatayud et al. (64)	No	Yes	Yes	No	No	Yes	Yes	Yes	No	Yes	Yes	7
McGill et al. (66)	No	Yes	Yes	No	No	Yes	Yes	Yes	No	Yes	Yes	7
Park et al. (67)	No	Yes	Yes	No	No	Yes	No	Yes	No	Yes	Yes	6
Lee et al. (68)	No	Yes	Yes	No	No	Yes	Yes	No	No	Yes	Yes	6
Anderson et al. (69)	Yes	Yes	Yes	No	No	No	Yes	Yes	No	Yes	Yes	7
Park and Yoo (70)	No	Yes	Yes	No	No	Yes	Yes	Yes	No	Yes	Yes	7
Snarr and Esco (71)	Yes	Yes	Yes	No	No	Yes	Yes	Yes	No	Yes	Yes	8
Kim et al. (72)	No	Yes	Yes	No	No	Yes	No	No	No	Yes	Yes	5
Sandhu et al. (4)	No	Yes	Yes	No	No	Yes	Yes	No	No	Yes	Yes	6
Marshall and Murphy (73)	Yes	Yes	Yes	No	No	Yes	Yes	No	No	Yes	Yes	7
Lehman et al. (21)	No	Yes	Yes	No	No	Yes	No	No	No	Yes	Yes	5
Freeman et al. (74)	Yes	Yes	Yes	No	No	Yes	Yes	Yes	Yes	Yes	Yes	9
